# Genomic regions and candidate genes linked with *Phytophthora capsici* root rot resistance in chile pepper (*Capsicum annuum* L.)

**DOI:** 10.1186/s12870-021-03387-7

**Published:** 2021-12-18

**Authors:** Dennis N. Lozada, Guillermo Nunez, Phillip Lujan, Srijana Dura, Danise Coon, Derek W. Barchenger, Soumaila Sanogo, Paul W. Bosland

**Affiliations:** 1 Department of Plant and Environmental Sciences, New Mexico State University, Las Cruces, NM 88003 USA; 2 Chile Pepper Institute, New Mexico State University, Las Cruces, NM 88003 USA; 3 Extension Plant Sciences, Plant Diagnostic Clinic, New Mexico State University, Las Cruces, NM 88003 USA; 4 World Vegetable Center, Shanhua, Tainan, 74151 Taiwan; 5 Department of Entomology, Plant Pathology and Weed Science, New Mexico State University, Las Cruces, NM 88003 USA

**Keywords:** Genotyping-by-sequencing, New Mexico RIL (NMRIL) population, QTL mapping, Phytophthora root rot, Single nucleotide polymorphisms

## Abstract

**Background:**

Phytophthora root rot, caused by *Phytophthora capsici,* is a major disease affecting *Capsicum* production worldwide. A recombinant inbred line (RIL) population derived from the hybridization between ‘Criollo de Morellos-334’ (CM-334), a resistant landrace from Mexico, and ‘Early Jalapeno’, a susceptible cultivar was genotyped using genotyping-by-sequencing (GBS)-derived single nucleotide polymorphism (SNP) markers. A GBS-SNP based genetic linkage map for the RIL population was constructed. Quantitative trait loci (QTL) mapping dissected the genetic architecture of *P. capsici* resistance and candidate genes linked to resistance for this important disease were identified.

**Results:**

Development of a genetic linkage map using 1,973 GBS-derived polymorphic SNP markers identified 12 linkage groups corresponding to the 12 chromosomes of chile pepper, with a total length of 1,277.7 cM and a marker density of 1.5 SNP/cM. The maximum gaps between consecutive SNP markers ranged between 1.9 (LG7) and 13.5 cM (LG5). Collinearity between genetic and physical positions of markers reached a maximum of 0.92 for LG8. QTL mapping identified genomic regions associated with *P. capsici* resistance in chromosomes P5, P8, and P9 that explained between 19.7 and 30.4% of phenotypic variation for resistance. Additive interactions between QTL in chromosomes P5 and P8 were observed. The role of chromosome P5 as major genomic region containing *P. capsici* resistance QTL was established. Through candidate gene analysis, biological functions associated with response to pathogen infections, regulation of cyclin-dependent protein serine/threonine kinase activity, and epigenetic mechanisms such as DNA methylation were identified.

**Conclusions:**

Results support the genetic complexity of the *P. capsici*–*Capsicum* pathosystem and the possible role of epigenetics in conferring resistance to Phytophthora root rot. Significant genomic regions and candidate genes associated with disease response and gene regulatory activity were identified which allows for a deeper understanding of the genomic landscape of Phytophthora root rot resistance in chile pepper.

**Supplementary Information:**

The online version contains supplementary material available at 10.1186/s12870-021-03387-7.

## Background

Phytophthora root rot, caused by the soil-borne oomycete *Phytophthora capsici*, is one of the most devastating diseases affecting *Capsicum annuum* (2n=2x=24) production across the globe [1–4]. Nearly a hundred years after *P. capsici* was first described by H. Leonian in New Mexico, USA in 1922 [5], the disease still remains a major problem in the state and worldwide causing an estimated annual loss of 100 million US dollars annually [6]. While using fungicides, crop rotation, and irrigation management can circumvent the effects of *P. capsici* in chile pepper [3], identifying sources of resistance alleles and using resistant cultivars remains the most eco-friendly and cost-effective approaches to minimize the effect of this pathogen [7, 8]. Identification and characterization of different *P. capsici* virulence groups are also important for breeding for disease resistance, as race-specific resistance has been previously reported [9–11].

Differences in patterns of inheritance for resistance to *P. capsici* have been demonstrated, imposing challenges on breeding for improved resistance in chile pepper. For example, a single dominant inheritance for resistance to *P. capsici* has been observed [12–15], whereas others have identified a two-gene model with dominant and recessive epistasis [16] as the mechanism of inheritance. Phytophthora root rot has distinct disease syndromes with each requiring different gene or genes for the expression of resistance [17]. The presence of a single dominant inhibitor gene affecting *P. capsici* resistance (*Ipcr*) in chile pepper has also been observed, where susceptibility of individuals cannot be explained by the absence of resistance genes, but, instead, results from the action of the *Ipcr* inhibitor interfering with the host-specific defense mechanism [18]. The *Phytophthora–Capsicum* pathosystem is more complex than what was initially thought, and breeding strategies that incorporate more diverse germplasm and novel genomics and sequencing approaches must be utilized to achieve local and global success in breeding for disease resistance [4].

The identification of quantitative trait loci (QTL) is one of the cornerstones of modern molecular plant breeding for genetic improvement of traits. A major step in QTL mapping involves the development of genetic linkage maps that indicate the position and relative genetic distances between markers along chromosomes or linkage groups, by genotyping a mapping population [19]. In genetic map construction, markers are first partitioned into linkage groups; then the correct order for these markers are identified, followed by the estimation of the genetic distances between adjacent markers [20]. After the development of the linkage maps, QTL analysis is implemented by using the phenotypic data collected from the individuals belonging to the mapping population. The choice of DNA-based markers, mapping functions, method of phenotypic data collection, mapping population type, and sample size for the development of linkage maps are among the factors affecting QTL identification and analysis [21–23]. Once QTL regions are identified, molecular marker development, validation, and fine mapping of genes can be implemented for marker-assisted selection (MAS) toward the genetic improvement of traits [24–26].

Characterization of resistance genes and DNA markers linked to these genes through MAS and QTL identification can result in the development of multiple and durable disease resistance [27]. Using biparental QTL mapping, several resistance loci have been identified in chile pepper, including *Phyt-1*, mapped on chromosome P7, *Phyt-2* (P1), and *Phyt-3* (P6), identified from a doubled-haploid population of ‘K9-11’ and ‘AC2258’ [28]. Another study mapped a locus, *Pc5.1* (P5), using recombinant inbred lines derived from the hybridization between ‘Criollo de Morelos-334’ (‘CM-334’) and ‘Early Jalapeno’ [29]. The QTL *Phyto5.2* [30]; and *QTL5.1*, *QTL5.2*, and *QTL5.3* [8], also on chromosome P5, were mapped by hybridizing ‘CM-334’ x ‘Yolo B’ and ‘CM-334’ x ‘ECW30R’, respectively. The use of genotyping-by-sequencing (GBS) for SNP marker discovery and genotyping has demonstrated a great potential for genomics-assisted breeding for the selection of lines with improved disease resistance. The GBS process involves a series of digestions using restriction enzymes, ligation, and PCR amplification steps, followed by the alignment of sequences to a reference genome [31, 32]. SNP markers are known for their flexibility, abundance in the genome, and ability to use standardized and scalable genotyping methods [33–35], and therefore have become a marker platform of choice for molecular plant breeding applications.

A population of recombinant inbred lines (RILs) was initially developed at New Mexico State University (New Mexico RIL or NMRIL) to study race structure in *Phytophthora capsici* [10]*.* The NMRIL population has been genotyped with 3,878 transcript-based single position polymorphisms distributed across the 12 chromosomes of chile pepper [36]. Currently, however, there are no existing GBS-SNP genetic maps for the NMRIL that can be used for QTL identification and MAS for improved disease resistance. Given the availability of genetic marker data, it would be relevant to examine the genetic landscape of *P. capsici* resistance for marker-assisted breeding towards improvement of disease resistance in current chile pepper germplasm. The present study aimed to develop a linkage map based on GBS-derived SNP markers and determine the genetic basis of *P. capsici* root rot resistance in chile pepper using QTL mapping and candidate gene analysis. Results rendered a better understanding of the genetic architecture of Phytophthora root rot which can facilitate breeding for improved *P. capsici* resistance in chile pepper.

## Results

### Disease ratings for the NMRIL

**Fig. 1:**
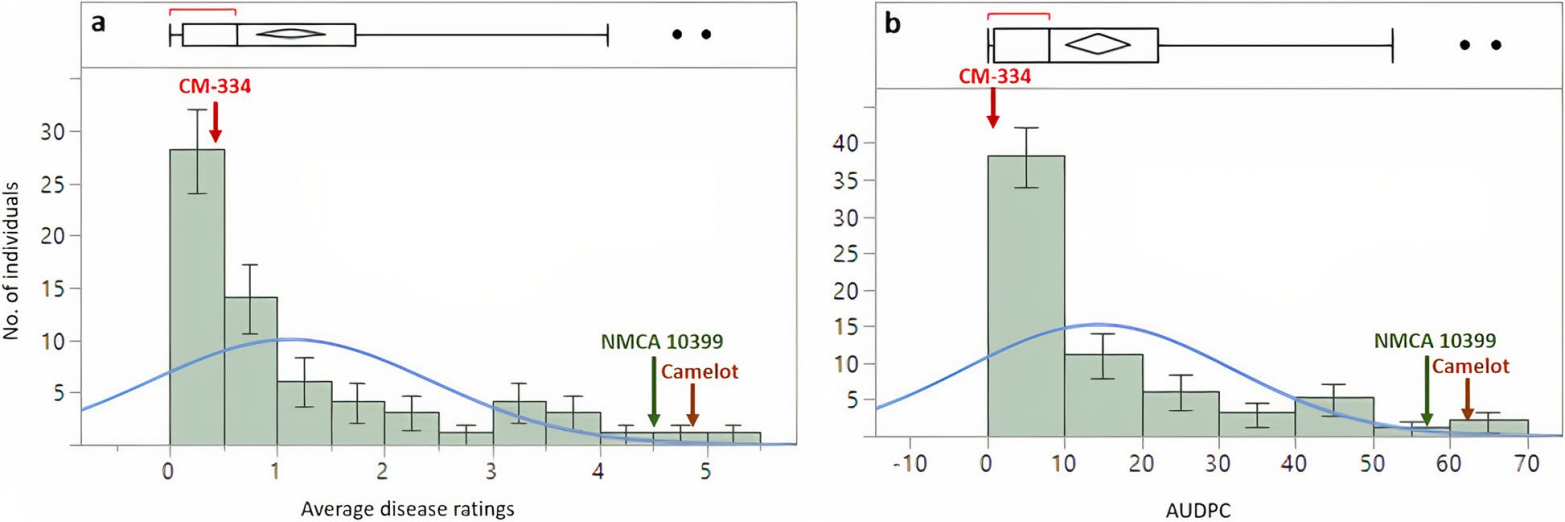
Distribution of mean disease scores (a) and area under disease progress curve (AUDPC) (b) from 2-14 days post-infection for the New Mexico RIL population inoculated with *Phytophthora capsici*. CM-334, NMCA 10399, and Camelot are the resistant and susceptible controls 1 and 2, respectively. Bars indicate standard errors. Blue solid curve represents the test for normality using the Shapiro-Wilk test

Analysis of variance (ANOVA) revealed significant differences between genotype, DPI, and block, whereas there were no significant differences between replications based on average disease severity scores (Additional file 1, Table S1)**.** A total of 49 NMRIL genotypes (74%) were not significantly different (Dunnett’s LSD, *P* > 0.05) with the average disease score for the resistant control (CM-334; 0.23); 15 of the 49 NMRILs had a mean disease rating of 0. Altogether, ten lines were moderately to highly susceptible to the *P. capsici* isolate. Eight lines did not differ significantly from the susceptible control 1 (NMCA 10399), whereas six lines were not significantly different from the susceptible control 2 (Camelot). No significant difference was observed between the susceptible controls in terms of disease ratings (NMCA 10399 (4.50) vs. Camelot (4.85)). Among the susceptible NMRIL lines, NMRIL_P had the highest mean disease severity score (5.0), followed by NMRIL_AX (4.73), and NMRIL_AAA (4.78). The distribution of mean disease scores was positively skewed toward resistance (Shapiro-Wilk test for normality, *P* < 0.0001; Fig. 1). Resistance to *P. capsici* had a moderately high broad-sense heritability at *H*^*2*^ = 0.57.

Disease symptoms of *P. capsici* infection began to manifest at 4 DPI for a number of the NMRILs and the susceptible controls. Five NMRIL lines across the two replications had necrosis and at least 50% defoliation, including NMRIL_P, which were dead (with a disease score of 6) at 4 DPI. NMRIL_P, NMRIL_AX, and NMRIL_AAA had the highest AUDPC values at 66, 61.94, and 52.5, respectively. Average AUDPC for the NMRIL population was 14.41. The resistant control, CM-334 had an AUDPC value of 2.55, whereas the susceptible controls, NMCA 10399 and Camelot had AUDPC of 59.29 and 63.75, respectively. Similar to the mean disease ratings, AUDPC values exhibited a positively skewed distribution (Shapiro-Wilk test for Normality, *P* < 0.0001; Fig. 1) toward resistance.

### Genome-wide SNP marker discovery using GBS

**Fig. 2:**
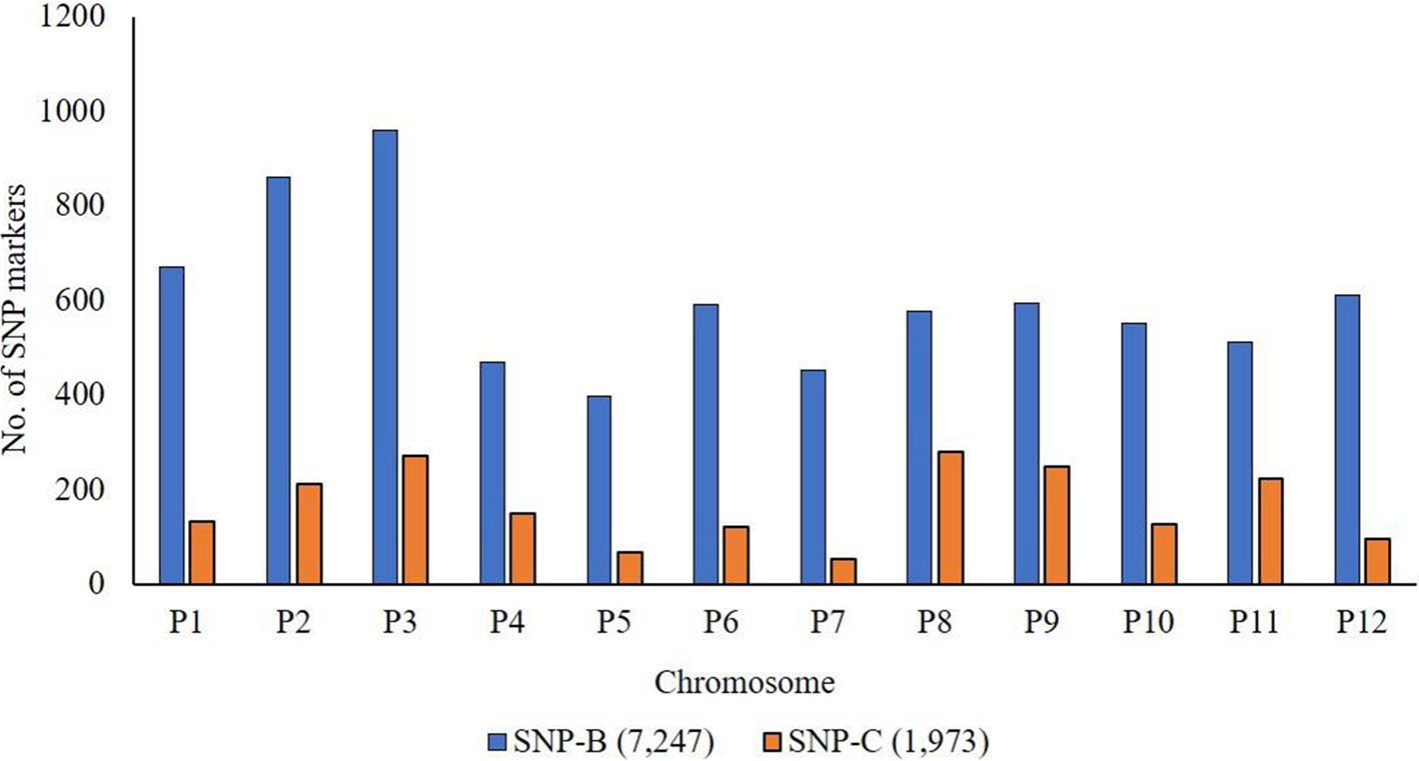
Distribution of SNP markers across the 12 chromosomes of chile pepper (*Capsicum annuum*). SNP-B set comprised of 7,247 markers polymorphic for the parental lines (CM-334 and ‘Early Jalapeno’). SNP-C set consisted of 1,973 polymorphic markers with no segregation distortion and was used to construct the linkage map

An average of 3,854,130 sequence reads across the whole genome were generated using the Illumina NovaSeq^TM^ 6000 next-generation sequencing platform for the parental lines and NMRIL population. A total of 75,839 SNP markers were discovered by aligning the sequences to the ‘Zunla-1’ reference genome; of which, 68,253 SNP markers (90%) (unfiltered; SNP-A set) with known positions were mapped, whereas 7,586 (10%) were unmapped and were excluded for further analysis. A total of 7,247 SNP loci (SNP-B set) polymorphic for the parental lines distributed across 12 chromosomes were discovered for the NMRIL population **(**Fig. 2**)**. The average frequency of minor alleles across the whole NMRIL population for the polymorphic markers (SNP-B set) was 0.31 and the proportion of heterozygous individuals was 0.04.

The most common nucleotide was adenine (A) (25.14%), followed by thymine (T) (24.1%), cytosine (C), (23.3%), and guanine (G) (23.10%) for the SNP-B set (Table 1). Across the SNP sites, 4.35% have ambiguous nucleotide calls. Overall, 4,029 sites (55.6%) had transitions, whereas 2,973 (41.0%) had transversion substitutions, where ‘C/T’ was the most observed substitution type (1,078; 14.9%) followed by ‘A/G’ (1,031; 14.2%). Among the transversions, ‘T/A’ and ‘A/C’ were the most common types across the SNP loci with 432 (6.0%) and 417 (5.8%), respectively. For the SNP-A (unfiltered) marker set, chromosomes P3, P1, and P2 had the highest number of markers with 9,459, 7,827, and 7,143 markers, respectively, whereas P11 (3,990), P9 (4,103), and P5 (4,254) had the fewest. Similarly, for the SNP-B set, chromosome P3 had the greatest number of SNP markers (960), followed by P2 (860), and P1 (671), whereas chromosomes P5, P7, and P4 had the least number of SNP loci discovered, with 399, 452, and 468 markers, respectively.

**Table 1:** Allele summary and substitution types represented by the identified polymorphic SNP markers (SNP-B set) for the New Mexico RIL population

Allele	Number of SNP sites	Frequency
A	125,689	0.25
T	120,652	0.24
C	116,487	0.23
G	115,443	0.23
Transition		
C/T	1,078	0.15
T/C	917	0.13
A/G	1,031	0.14
G/A	1,003	0.14
Transversion		
T/A	432	0.06
A/T	399	0.06
A/C	417	0.06
C/A	397	0.05
T/G	402	0.06
G/T	388	0.05
G/C	274	0.04
C/G	264	0.04

### Genetic map construction

**Fig. 3:**
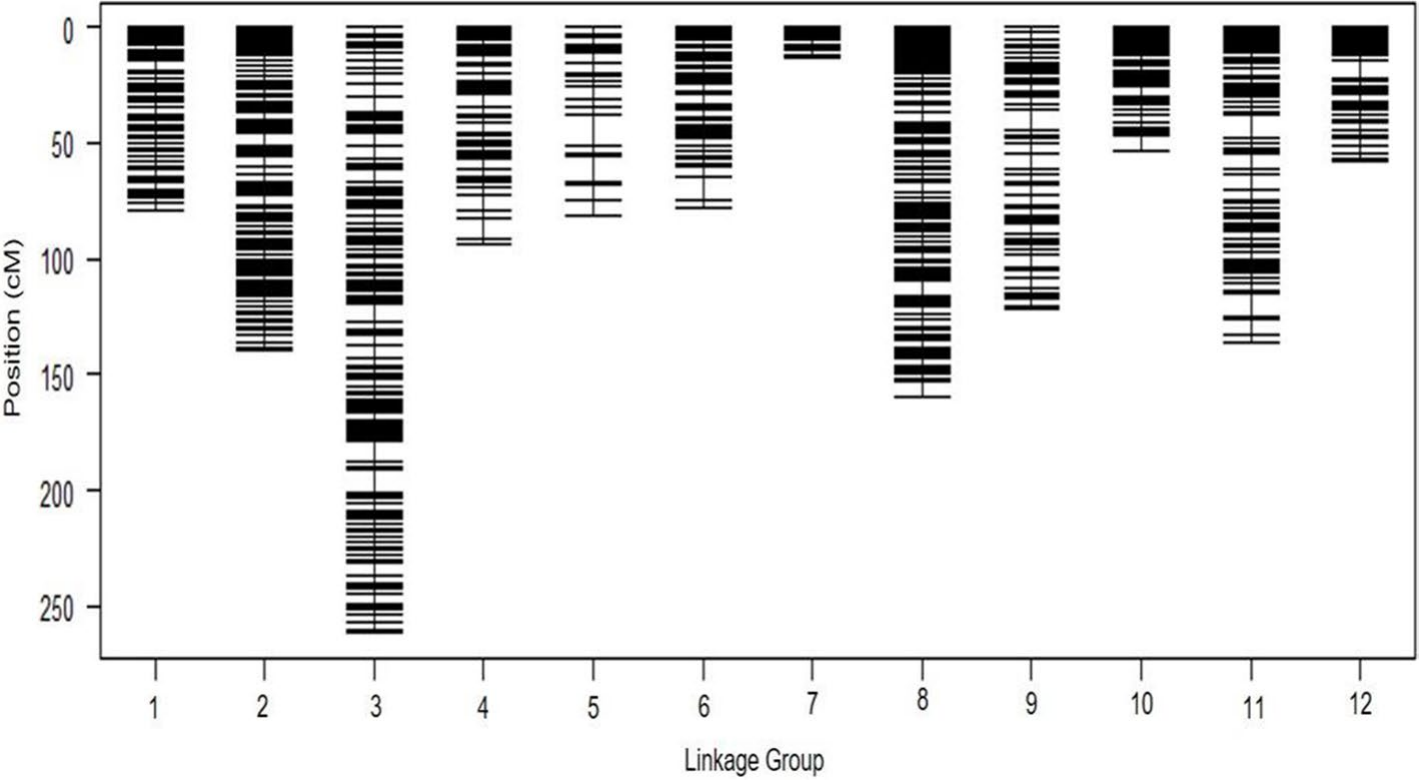
SNP-based genetic linkage map for the New Mexico Recombinant Inbred Line (NMRIL) population using 1,973 markers. Black bars indicate SNP marker loci

A total of 5,274 (72.78%) markers significantly (*P* < 0.05) deviated from 1:1 Mendelian segregation ratio and were excluded for the development of genetic linkage map. The remaining 1,973 SNP markers (SNP-C set) exhibiting no segregation distortion was used for linkage map construction (Additional file 1, Table S2). Genetic map construction resulted in 12 linkage groups corresponding to the 12 chromosomes of chile pepper*,* with a total length of 1,277.7 cM and marker density of ~1.5 SNP/cM (Table 2; Fig. 3). Across the different linkage groups, LG8 was comprised of the highest number of SNP markers (279), followed by LG3 (271), and LG9 (248). In contrast, LG7, LG5, and LG12 had the least number of markers with 54, 66, and 94, respectively. The proportion of markers exhibiting no segregation distortion was highest for LG8 (48.3%), followed by LG11 (43.4%), and LG9 (41.8%). The total length (in cM) of the LG ranged from 13.4 (LG7) to 261.7 (LG3). In terms of frequency of SNP markers per cM, LG7 (4.0), LG10 (2.3), and LG9 (2.0) had the highest frequency, whereas LG5, LG3, and LG2 had the lowest, with 0.81, 1.0, and 1.54 SNP/cM, respectively.

**Table 2:** Summary of SNP markers for the *C. annuum* linkage groups

LG^a^	Total no. of polymorphic markers	Length (Mb)	No. of markers without SD^b^	Markers without SD (%)	Length (cM)	Freq. of SNP (per cM)	Gap between adjacent SNP (max; cM)	Gap between adjacent SNP (ave; cM)	*R*^*2* c^
1	671	300.79	131	19.5	79.6	1.6	4.2	0.61	0.19
2	860	163.94	213	24.8	139.7	1.5	6.1	0.66	0.12
3	960	261.45	271	28.2	261.7	1.0	10.2	0.97	0.72
4	468	215.67	148	31.6	94.2	1.6	8.7	0.64	0.004
5	399	217.20	66	16.5	81.2	0.8	13.5	1.23	0.10
6	592	219.19	121	20.4	78.3	1.5	9.9	0.65	0.33
7	452	222.07	54	12.0	13.4	4.0	1.9	0.25	0.01
8	578	153.27	279	48.3	159.6	1.7	7.2	0.57	0.92
9	593	238.74	248	41.8	121.9	2.0	9.2	0.49	0.53
10	552	205.61	126	22.8	53.9	2.3	7.6	0.43	0.06
11	512	219.21	222	43.4	136.1	1.6	9.7	0.61	0.35
12	610	229.92	94	10.3	58.1	1.6	7.7	0.62	0.03
Total	7,247^d^	2,647.06^e^	1,973^f^		1,277.7^g^				

^a^Linkage group

^b^SD- segregation distortion

^c^Coefficient of collinearity; represented as the *R*^*2*^ value for the regression analysis with genetic distance (cM) as a linear function of the marker order for the physical locations of each SNP locus

^d^Total number of SNP markers for the SNP-B set

^e^Total length of map for SNP-B set (in Mb)

^f^Total number of SNP markers for the SNP-C set which was used for the construction of linkage map

^g^Total length of map for SNP-C set (in cM)

The maximum intervals (gaps) between consecutive SNP loci ranged between 1.9 (LG7) to 13.5 cM (LG5). Average gap between adjacent markers was highest for LG5 (1.23 cM), LG3 (0.97), and LG2 (0.66) and was lowest for LG7 (0.25), LG10 (0.43), and LG9 (0.49). Average marker interval across all the LG was 0.65 cM. Across the different LG, the coefficients of collinearity *R*^*2*^ between marker order number based on the physical map and cM distances in the linkage map were highest for LG8 (0.92), followed by LG3 (0.72), and LG9 (0.53). Conversely, *R*^*2*^ values were lowest for LG4, LG7, and LG12 with 0.004, 0.01, and 0.03, respectively.

### QTL and candidate genes for *P. capsici* resistance

**Fig. 4:**
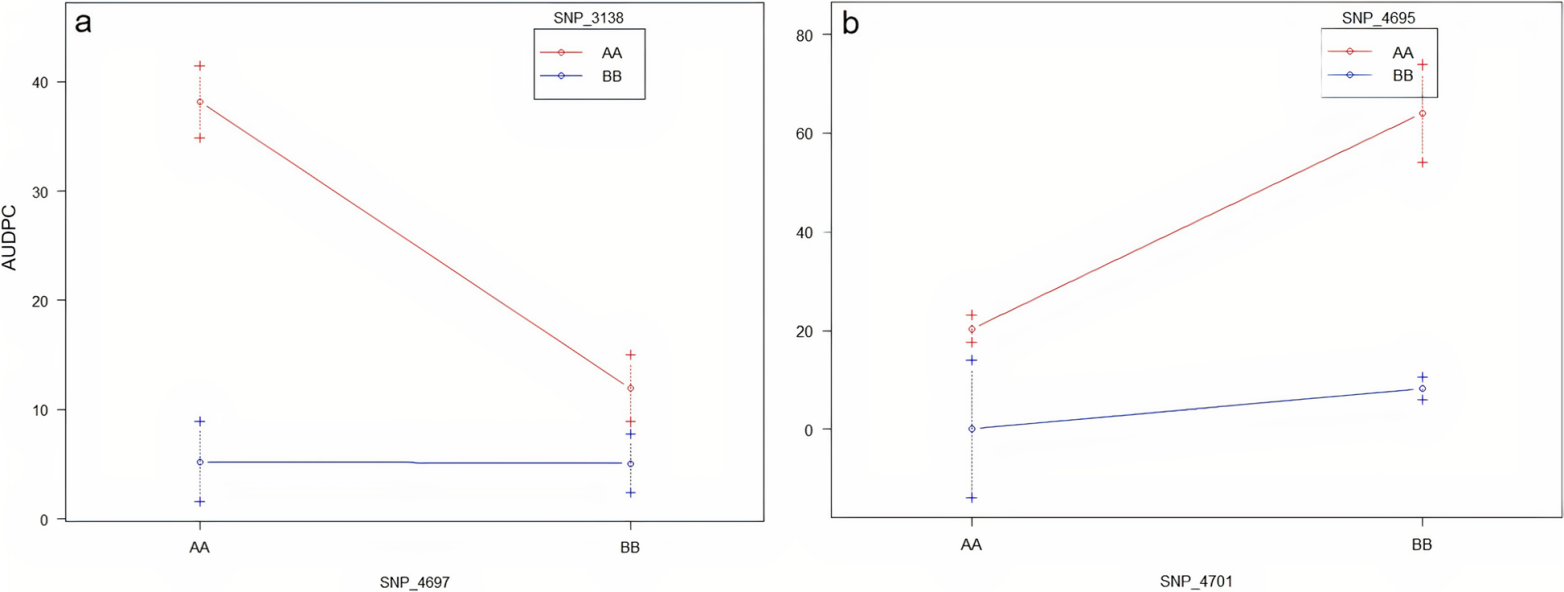
Effect plots for the *Phytophthora capsici* resistance QTL identified on chromosomes P5 and P8 showing additive interactions. SNP_3138 (*SCM002816.1_34981103*; chromosome P5) showed additive interaction with SNP_4697 (*SCM002819.1_132653758;* chromosome P8) whereas SNP_4695 (*SCM002819.1_13265375*; chromosome P8) demonstrated additive interaction with SNP_4701 (*SCM002819.1_133538774*; chromosome P8) (Table 2). AA allele = CM-334; resistant parent; BB allele= Early Jalapeno, susceptible parent)

Main effect QTL associated with *P. capsici* root rot resistance (AUDPC), designated as *QTL.Pc5*, *QTL.Pc8.1*, and *QTLPc.9*, were identified in chromosomes P5, P8, and P9 of chile pepper, respectively (Table 3). These QTL were related with 19.7 (*QTL.Pc8.1*) to 30.4% (*QTL.Pc5*) of phenotypic variation for resistance to *P. capsici*, with LOD scores ranging between 3.10 and 5.20. The LOD value for declaring a significant QTL was calculated at 2.95 for a threshold of α = 0.10. There were two pairs of QTL regions identified using multiple QTL mapping approach on chromosomes P5 and P8, where *QTL.Pc5* and *QTL.Pc8.1* showed evidence of additive effects (*P* = 0.001) and was related to 55.7% of phenotypic variation for *P. capsici* resistance (Fig. 4). Another pair of QTLs in chromosome P8, *QTL.Pc8.1* and *QTL.Pc8.2*, also demonstrated interaction (*P* = 0.014) with an LOD score of 6.5 and was associated with 35.8% of variation for disease resistance.

**Table 3:** Genomic regions associated with *Phytophthora capsici* root rot resistance represented as the area under disease progress curve (AUDPC) for the New Mexico RIL (NMRIL) population

QTL	Peak SNP marker	QTL Model	Chr.	Location (Mb)	Location (cM)	LOD	1.5 LOD interval (cM)	Percent variation explained
*QTL.Pc5*	*SCM002816.1_34981103*	*y = Q*	P5	34.98	81.2	5.20	76.0-81.2	30.4
*QTL.Pc8.1*	*SCM002819.1_132653758*	*y = Q*	P8	132.65	82.9	3.10	82.93-94.0	19.7
*QTL.Pc9*	*SCM002820.1_235989419*	*y = Q*	P9	235.99	18.0	3.40	18.0-29.76	21.0
*QTL.Pc5* x *QTL.Pc8.1*	*SCM002816.1_34981103; SCM002819.1_132653758*	*y = Q1 + Q2* + *Q1 x Q2*	P5_P8	34.98_132.65	81.2_82.9	11.68^f^ (8.14^ad^)	76.0-94.0	55.7 (43.3^ad^)
*QTL.Pc8.1* x *QTL.Pc8.2*	*SCM002819.1_13265375; SCM002819.1_133538774*	*y = Q1 + Q2* + *Q1 x Q2*	P8_P8	132.65_133.54	82.9_78.0	6.35^ad^	82.93-94.0	35.8^ad^

^ad^Additive effect model. Unless otherwise indicated, the percent variation explained represents the values for the full QTL model

A total of 152 candidate genes were identified using the flanking sequences for the peak SNP marker loci for the QTLs identified on chromosomes P5, P8, and P9 (Additional File 1, Table S3). There were 76 candidate genes identified for chromosome P5, whereas 46 and 30 candidate genes were predicted for chromosomes P8 and P9, respectively. Analysis of candidate genes revealed predicted functions related to various biological processes including regulation of cyclin-dependent protein serine/threonine kinase activity, cell cycle and division, cell wall organization and biosynthesis, ADP/ATP transport, and lipid and carbohydrate metabolism, among others in *C. annuum*. Similarly, predicted functions related to serine/threonine kinase activity, cell division, and cell cycle were identified for candidate genes orthologous to those in eggplant (*S. tuberosum*). Several other candidate genes were implicated for epigenetic mechanisms such as regulation of DNA methylation to orthologs of tobacco (*N. attenuata*) and histone H2A K63-linked deubiquitination in tomato (*S. lycopersicum*). Candidate genes orthologous to those in *Arabidopsis* were identified with predicted functions related to defense response to fungus, chromatin organization and silencing, and histone H3-K27 methylation.

## Discussion

Phytophthora root rot, caused by *P. capsici*, remains one of the most devastating diseases affecting chile pepper production worldwide. In the past, molecular breeding has benefited the development of disease resistant chile pepper varieties. Marker-assisted selection through QTL identification is one of the approaches that can facilitate genetic improvement for increased *P. capsici* resistance. In the current study, GBS-SNP markers were used to develop linkage map and a QTL mapping approach was implemented to identify genomic regions and candidate genes related to Phytophthora root rot resistance using a RIL population of chile pepper. Recombinant inbred populations are valuable genetic resources for the dissection of the genetic architecture of complex traits with agricultural and economic importance. Some advantages of using RIL for linkage map construction and genetic mapping include: (1) each line can be propagated eternally and needs to be genotyped only once; (2) multiple individuals from each line can be phenotyped to decrease variation due to genotype, environment, and trait data collection; and (3) greater mapping resolution can be achieved as the breakpoints in each line are denser than those that occur in any single meiotic event [37].

### A GBS-SNP based genetic linkage map for chile pepper

Construction of a genetic map for the NMRIL population resulted in the identification of the number of linkage groups corresponding to the haploid chromosome number for chile pepper (*n*=12) with a total genetic distance of 1,277.7 cM. The map presented herein had a comparable length with the intraspecific map previously developed by Hill et al. [36] spanning a total of 1,399 cM for the NMRIL using a transcript-based GeneChip^®^ technology. The LG7 had the fewest number of total markers consistent with what was previously observed, indicating a reduced polymorphism on LG7 between the intraspecific parental lines for the NMRIL [36]. Notable differences between the Hill et al. map and the present GBS-SNP map for the NMRIL include the distribution of SNP loci on each group, lengths of each LG, and the maximum interval (gaps) between adjacent markers. These apparent differences between the linkage maps could be a consequence of the marker platform used for genotyping the population. Among all the groups, LG3 had the longest length in agreement with a genetic map developed recently using specific locus amplified fragment sequencing (SLAF-seq) markers [38]. The current GBS-SNP based map for the NMRIL had a shorter length compared to several linkage maps previously constructed for chile pepper [8, 39–41], and was longer relative to that of an intraspecific linkage map for *C. baccatum* [42]. The genotype data (SNP-C set) used for linkage map construction had a proportionate AA and BB genotypes (47.4 and 49.7%, respectively) across the NMRIL, consistent with previous observations by Rehrig et al. [29] when the same population was genotyped using 3,887 contig markers derived from the Pepper Affymetrix Chip platform [43].

### Race specific resistance or susceptibility imposes a challenge to *P. capsici* resistance breeding

The distribution of the NMRIL population was positively skewed for the mean disease scores and AUDPC values indicating that most of the genotypes were resistant and can be used as sources of resistant alleles against *P. capsici.* The majority (74%) of the lines screened exhibited no significant difference from the CM-334 (resistant control) in terms of average disease ratings. Race-specific resistance or susceptibility for some of the NMRIL genotypes was demonstrated in our study. For example, the NMRIL_H line displayed no visible disease symptoms with a mean disease rating of 0 after 14 DPI but was previously found to be susceptible to eight other isolates of *P. capsici* from New Mexico, USA [44]. NMRIL_P, a highly susceptible RIL to the ‘PWB-185’ isolate used in this study with a mean disease score of 5.0 and AUDPC value of 66 had high levels of resistance to 13 different *Phytophthora* isolates from California, New Mexico, and the Netherlands, including the ‘PWB-80’, a virulent strain [10] and against 24 isolates in Taiwan [11]. The presence of race specific resistance or susceptibility to different *Phytophthora* isolates imposes a challenge in breeding for resistance. The use of QTL mapping and molecular marker development enables breeders to facilitate the development of chile pepper varieties that are resistant to most if not all of the isolates present in a certain geographic area through marker-assisted gene pyramiding. The NMRIL population, overall, remains an indispensable genomic resource to study resistance to different *Phytophthora* isolates and to identify QTL affecting resistance to this important disease.

### Major QTL linked with ***P. capsici*** resistance were identified in chromosomes P5, P8, and P9

Genomic regions related to disease resistance to the *P*. *capsici* isolate ‘PWB-185’ were identified in key chromosomal regions in the chile pepper genome, including chromosomes P5, P8, and P9. Chromosome P5 has been previously identified as a major chromosome harboring QTLs and genes related to *P. capsici* resistance in chile pepper [15, 29, 38, 45]. In the current study, a QTL designated as *QTL.Pc5,* had an LOD score of 5.20 and was responsible for ~30% of variation for *P. capsici* root rot resistance. The *QTL.Pc5* with peak SNP marker located at 34.98 Mb coincided with three previously identified QTL namely, *E1Kpc-5.3*, *E2KPc-5.3*, and *E2My-5.3,* using a RIL population derived from CM-334 (resistant) and ECW30R (susceptible) mapped at 34.6-37 Mb by Siddique et al. [8]; these could be the same QTL region identified for *P. capsici* resistance on the short arm of chromosome P5. Moreover, *QTL.Pc5* lies ~2.98 Mb to the *Pc5.1* QTL previously mapped by genotyping the NMRIL using a Pepper Affymetrix chip [29]. Liu et al. [15] mapped another major QTL related to *P. capsici* resistance, *Phyto5SAR* at ~2.06 Mb, using bulk segregant analysis in chile pepper; albeit located further upstream of the *QTL.Pc5* identified in the current study, these results overall still confirmed the presence of a large QTL region for *P. capsici* resistance in the short arm of chromosome P5. The *Pc5.1* QTL region [46] was recently expanded by anchoring genetic markers into the pepper genomic sequence resulting in an extended *Pc5.1* (*Ext-Pc5.1*) mapped at 8.35-38.13 Mb [47]. Previous meta-QTL analyses further confirmed the role of chromosome P5 in conferring resistance to *P. capsici* in pepper. Mallard et al. [46] identified meta-QTL namely, *MetaPc5.1*, *MetaPc5.2,* and *MetaPc5.3* with confidence intervals ranging from 2.21 (*MetaPc5.1*) to 4.61 cM (*MetaPc5.3*) related to broad-spectrum resistance. More recently, four meta-QTL (MQTL) regions associated with *P. capsici* resistance in chromosome P5, two of which were delimited to a confidence interval of <1.0 cM [48]. Notably, the *QTLPc.5* identified in the current study was located at a proximal distance (~6 cM) to the *MQTL5.3* identified recently [49]. Altogether, our results combined with previous studies further established the importance of chromosome P5 as a major genetic hotspot for QTL related to *P. capsici* resistance in chile pepper. The co-localization of major *P. capsici* resistance QTL in chromosome P5 can ultimately direct molecular breeding and selection decisions for improving disease resistance in chile pepper. However, accurate QTL identification and molecular marker development on chromosome P5 has been a major challenge as this chromosome is saturated with nucleotide-binding site leucine-rich repeat (NBS-LRR) genes with high levels of duplication resulting from transposable elements [49, 50].

Evidence for additive effect interactions for two pairs of QTLs in chromosomes P5 and P8 contributing to 35-55% of phenotypic variation were identified (Fig. 4). Interaction of QTL for *P. capsici* resistance have been well established in previous studies in chile pepper [45, 51–53], and indicates the potential of combining favorable alleles to improve disease resistance. In other crops such as winter wheat (*Triticum aestivum* L.), pyramiding resistant alleles has been demonstrated to increase resistance or tolerance to various diseases such as stripe rust (causative agent: *Puccinia striiformis* [54], eyespot disease (*Oculimacula yallundae* and *Oculicmacula acuformisi*) [55]; and snow mold (*Microdochium* [*Fusarium*] *nivale*) [56]. The QTL interactions identified in the current study have not been reported elsewhere, and hence could represent novel genomic regions showing additive effects that could be targeted for future breeding and selection for improved *P. capsici* resistance. It is interesting to note that the source of the resistant allele for the P5 and P8 additive QTL was the susceptible parent, Early Jalapeno (Fig. 4a); in contrast to the P8 QTL where the CM-334 (resistant parent) was the source of the favorable allele (Fig. 4b). Although considered as the less common source of favorable alleles, Early Jalapeno was previously identified to be the contributing parental line for the resistant alleles for 3 and 5-day post infection for Phytophthora fruit rot resistance in chile pepper [57].

### Candidate genes reflect the genetic complexity of *P. capsici* resistance

Analysis of candidate genes using flanking sequences for the peak SNP marker for *QTL.Pc5* in chromosome P5 revealed a wide range of putative biological functions related to defense response to fungi and bacteria, epigenetic mechanisms such as DNA methylation, histone deubiquitination, and chromatin silencing/remodeling; cell cycle and division, and DNA repair, among others demonstrating the complex nature of *P. capsici* resistance in chile pepper. The QTL in chromosome P5 has been demonstrated as a major genomic region harboring disease resistance (R) genes from various gene families. For instance, two R genes with NBS-LRR resistant structures were identified and considered as strong candidate genes for Phytophthora resistance on chromosome P5 [15]. The LRR regions bind to extracellular ligands and cytoplasmic kinase domains for phosphorylation-related signal transduction, either by direct interaction with the effectors released by the pathogen or by monitoring the host proteins targeted by these effectors [58, 59]. Candidate genes involved in the regulation of cyclin-dependent protein serine/threonine kinase activity were also identified in the present work, consistent with the genes with LRR receptor-like serine/threonine protein kinase activity previously implicated for *P. capsici* disease resistance [8]. The peak SNP marker *SCM002816.1_34981103* (34.98 Mb) for *QTL.Pc5* was located in proximity (~0.40 Mb) with the candidate gene, *T459_14320* (35.39-35.40 Mb) involved in the regulation of cyclin-dependent protein serine/threonine kinase activity in *C. annuum*. Moreover, this peak SNP locus was within the *Ext-Pc5.1* QTL region (8.35-38.13 Mb in chromosome P5) which harbors 14 NBS-ARC (*APAF-1*, R proteins, and *CED4* domain) genes identified recently by Du et al. [47], indicating that the marker itself is located near or within coding regions. It might be difficult to tell precisely if these peak SNP markers are associated with any structural changes; nonetheless, in the case of *SCM002816.1_34981103*, we could infer possible structural changes in the candidate genes as the SNP itself possesses a transversion substitution (‘T/G’), which involves exchange of one-ring or two-ring structures. In another study, a downy mildew (*Peronospora tabacina*) resistance gene, *CaDMR1* encoding a homoserine kinase, was also observed to be highly associated with a major pepper QTL, *Pc5.1*, on chromosome P5 [29]. A primary challenge for pathogens is to breach the host cell wall, which offers formidable and dynamic barriers while avoiding detection by immune receptors [60]. Candidate genes with functions related to plant cell wall biogenesis and organization were also identified in the current study, indicating the relevance of these physical barriers in the cell as a defense against infections by *P. capsici*. There has been evidence for the possible role of epigenetic mechanisms on resistance to *P. capsici* based on recent transcriptomic and meta-QTL analyses [47, 48], and our results support the possibility for epigenetics’ function in conferring Phytophthora root rot resistance. It would be relevant, therefore, to dissect the epigenome of chile pepper to gain a deeper understanding of the mechanism for resistance to Phytophthora root rot. Overall, the diversity of the functions identified for the candidate genes demonstrate the complex nature of *P. capsici* resistance in chile pepper.

## Conclusions

A QTL mapping approach was implemented to dissect the genetic architecture of *Phytophthora* *capsici* root rot resistance using a chile pepper RIL population. Major QTL in key chromosomal regions were identified for *P. capsici* resistance. Chromosome P5 was established as a primary genetic hotspot containing disease resistance QTL. Candidate gene analysis revealed putative gene functions associated with biological processes including defense response, DNA repair, regulation of kinase activity, and epigenetic mechanisms such as DNA methylation and chromatin remodeling. The relatively low number of lines used for genetic mapping in the current study, nonetheless, would warrant future QTL validation studies using different chile pepper breeding populations. Kompetitive allele specific markers (KASP^®^) [61] will be ultimately developed for QTL validation, marker-assisted breeding, molecular cloning, and genome-wide selection to improve *P. capsici* resistance in chile pepper.

## Materials and methods

### Plant population

In the current study, an F_7_ recombinant inbred line (RIL) population derived from the hybridization between ‘Criollo de Morellos’ (CM-334), a resistant landrace from Mexico, and ‘Early Jalapeno’, a susceptible line ([10]; New Mexico RIL, NMRIL) was screened for resistance to Phytophthora root rot. A single seed descent procedure was used to develop the population. In total, 66 NMRIL lines were evaluated for resistance to *P. capsici* root rot. The NMRIL was initially used to characterize different races of *P. capsici* causing Phytophthora root rot to establish a stable host differential for the *Phytophthora*–*Capsicum* pathosystem [10]. The NMRIL has been screened for disease resistance using different isolates of *P. capsici* from different geographical regions of the world, including Argentina, the Netherlands, Turkey, and the USA [11, 44, 62, 63].

### Production of *P. capsici* zoospores

Zoospores of *P. capsici* were produced as described in previous works [1, 64]. Isolate ‘PWB-185’ was grown for 5 days on V8-agar medium amended with different antibiotics, namely, rifampicin, ampicillin, pimaricin. Plugs (1 cm diameter) were cut off the culture of *P. capsici* using a cork borer under a fume hood. Five plugs were transferred to a petri dish containing 25 mL of sterile distilled water. Ten plates were prepared and then stored for 48 hours in an incubator at 25°C under fluorescent light for sporangia formation. Then, to induce zoospore release, petri dishes were transferred to a 4°C cold room for 30 min and returned to the 25°C incubator for another 30 min. The contents of the petri dishes were filtered through a cheesecloth into an Erlenmeyer flask to collect the zoospores. Total zoospore concentration in the suspension was estimated using a hemocytometer (Hausser Scientific, USA). The concentration of the suspension was adjusted with sterile distilled water to yield ~2,000 zoospores per mL.

### *P. capsici* inoculation and disease scoring

Seeds of each NMRIL were planted in F1020 standard insert multi-cell trays (American Horticultural Supply Inc., CA, USA) at the Fabián García Research Center, New Mexico State University, Las Cruces, NM and were grown under standard greenhouse conditions [65]. At the 5-8 leaf stage, seedlings were soil-inoculated by dispensing 5 mL of the 2,000 zoospore per mL into each cell, resulting in ~10,000 *P. capsici* zoospores of ‘PWB-185’ isolate per cell. Each NMRIL has four cells with up to four seedlings (1 seedling per cell) per replication [1]. Starting at two days post infection (DPI), disease severity was scored every two or three days for each individual seedling using a scoring system from 0-6, with 0 = no visible disease symptoms, 1 = stem necrosis with no girdling, 2 = stem necrosis with girdling, 3 = stem necrosis with less than 50% defoliation, 4 = stem necrosis with greater than 50% defoliation, 5 = wilted, and 6 = dead [64, 66]. Disease severity ratings were recorded to a maximum of 14 DPI. The disease severity data was used to compute the area under disease progress curve (AUDPC) for each NMRIL as described below.

### Experimental design and statistical analyses

A randomized complete block design with two replications was used in screening the NMRIL population for *P. capsici* resistance. Each NMRIL consisted of a set of up to four plants, whereas each block consisted of 15 NMRIL and one resistant and two susceptible controls, namely, CM-334 (Resistant), NMCA 10399 (Susceptible 1), and ‘Camelot’ (Susceptible 2). Area under disease progress curve for each NMRIL was calculated using the ‘audpc’ function in the ‘agricolae’ [67] package in R [68] using the formula: $\sum_{i=1}^{n-1}\frac{{y}_{i}+{y}_{i+1}}{2} \mathrm{x} ({t}_{i+1}- {t}_{i})$, where *y*_*i*_ is score of plants (0–6) at the *i*^th^ observation (*i* = 1 being the first observation at time zero), *t*_*i*_ is the number of days post-infection at the *i*^*th*^ observation; and *n* is the number of observations.

Analysis of variance (ANOVA) was implemented using a standard least square linear model with the restricted maximum likelihood (REML) approach in JMP 13.2.1 [69] where entry, DPI, block, and replications were considered as fixed effects. The NMRIL lines were compared with the resistant and susceptible checks using Dunnett’s test. Test for normality of disease score distribution was implemented using the Shapiro-Wilk Test [70] in JMP 13.2.1. Broad-sense heritability for *P. capsici* resistance was calculated using the formula: *H*^*2*^ = σ^2^_G_/ (σ^2^_G_ + σ^2^_E_), where *σ*^*2*^_*G*_ is the variance due to genotype and σ^2^_E_ is the variance due to residual.

### DNA extraction and quantification

Using Qiagen Collection microtubes, leaf tissues were collected from 30 to 45-day-old chile pepper seedlings grown in standard insert multi-cell trays. Approximately 50 mg of fresh leaf samples were extracted using Qiagen DNEasy^®^ plant extraction kits through the University of Minnesota Genomics Center DNA extraction facility (https://genomics.umn.edu/service/dna-extraction). Picogreen (Thermofisher Scientific, MA, USA) quantified the DNA, and samples were normalized to 10 ng/ul for GBS.

### Library preparation and genotyping using GBS

Genotyping-by-sequencing (GBS) for the NMRIL population was conducted through the University of Minnesota Genomics Center (https://genomics.umn.edu/services/gbs) using a single-enzyme digestion protocol described previously [71]. Briefly, a total of 100 ng of DNA was digested with 10 units of *ApeKI* restriction enzyme (New England Biolabs, Inc., MA, USA) and incubated at 75°C for 2 hours, and heat inactivated at 80°C for 20 minutes. The DNA samples were ligated with 200 units of T4 ligase (New England Biolabs, Inc. MA, USA) and phased adaptors with the -CWG overhang at 22°C for 1 hour and heat inactivated. The ligated samples were purified with solid phase reversible immobilization beads and then amplified for 18 cycles with 2x NEB Taq Master Mix to add the barcodes. The GBS libraries were then purified, quantified, and pooled. Fragments with the 300-744 bp size region were selected and diluted to 1 nM for sequencing on the Illumina NovaSeq 6000 (Illumina, CA, USA) using single end 1×100 reads.

The raw FASTQ files were demultiplexed using the Illumina ‘bcl2fastq’ software (Illumina, CA, USA) and were aligned to the ‘Zunla-1’ reference genome (GCA_000710875.1, PRJNA186921, v.1.0; https://www.ncbi.nlm.nih.gov/nuccore/ASJU00000000.1/) [72] using the Burrows-Wheeler Aligner [73]. The raw variant call format (VCF) files were processed using VCFtools to remove variants with minor allele frequency <1%, genotype rates <95%, and samples with genotype rates <50%. Imputation of missing data was implemented using the linkage disequilibrium *k*- nearest neighbor joining genotype imputation (LD-kNNi) [74]. The VCF files were converted to HapMap and ABH formats using TASSEL 5.2.67 [75].

### Genetic map construction and QTL mapping

Development of the genetic linkage map used the ‘mstmap.cross’ function of the ‘ASMap’ package [76] in R that implements the ‘minimum spanning tree’ map (MSTmap) algorithm [20] using a ‘maximum likelihood’ objective function, a genetic distance threshold of 15 cM, and *P*-value of 1×10^-08^ using 66 NMRIL lines. SNP markers showing significant deviation from 1:1 Mendelian segregation ratio (Chi-square, χ^2^ goodness-of-fit test; *P* < 0.05) were identified and excluded for the development of the linkage map. Genetic distances were calculated using the ‘Kosambi’ mapping function that corrects crossover interference considering that one crossover prevents another nearby [77]. Coefficients of collinearity (*R*^*2*^) between the physical and genetic maps for each LG were calculated by regressing the cM distances as a linear function of the marker order positions of the physical map [36] using a bivariate fit approach in JMP 13.2.1. Marker distances on chromosomes were visualized using the ‘chromoMap’ [78] and ‘R/qtl’ [79] packages in R.

Identification of QTL was conducted using the Haley-Knott regression interval mapping approach [80] in the ‘R/qtl’ package in R. First, a single-QTL genome scan was performed, then a 1,000-permutation test determined the logarithm of the odds (LOD) score in declaring a significant QTL [81] at a threshold of α = 0.10 tail of the null distribution. Two-dimensional, two-QTL, and multiple-QTL mapping strategies were conducted to identify additive and epistatic effects after detecting single significant QTL. A multiple interval mapping pipeline was used to calculate QTL effects, refine positions, and estimate variance explained of QTL models. Confidence intervals for each QTL were identified using the 1.5-LOD support interval function in R/qtl.

### Candidate gene mining

Flanking sequences for the peak markers for the identified QTL were compared against the genome of ‘Criollo de Morellos 334’ (‘CM-334’; Genome assembly (GA): ASM512225v2) (*C. annuum*) using the BLASTn function in EnsemblPlants [82] (https://plants.ensembl.org/index.html; accessed on 06 September 2021) using an E-value cut-off of 10 and a ≥ 95% sequence identity. Annotated genes (within 0.50 Mb of the peak SNP locus) and their biological functions were listed. Further, orthologous genes from other solanaceous crops including potato (*S. tuberosum* DMI-3 516 R44; GA: SolTub_3.0), tomato (*Solanum lycopersicum* cv. Heinz 1706; GA: SL3.0), and wild tobacco (*Nicotiana attenuata* coyote tobacco; GA: NIATTr2), and *Arabidopsis thaliana* (GA: TAIR10) were identified based on biological functions using a gene ontology-based sequence annotation approach in EnsemblPlants.

## Supplementary Information

**Additional file 1: Table S1. **Analysis of variance for average disease severity ratings for *Phytophthora capsici* resistance for the New Mexico RIL population. **Table S2. **Genetic and physical positions of the 1,973 SNP markers used for the construction of genetic linkage map.** Table S3. **Candidate genes and their potential biological functions for QTL related with *P. capsici *resistance in chile pepper.

## Data Availability

The datasets generated and analyzed during the current study are available from the corresponding author on reasonable request.

## Abbreviations

GBS: genotyping-by-sequencing
NMRIL: New Mexico recombinant inbred line
QTL: quantitative trait loci
RIL: recombinant inbred line
SNP: single nucleotide polymorphisms
